# 改良的内切酶突变体富集法检测肺癌标本中*EGFR*基因突变

**DOI:** 10.3779/j.issn.1009-3419.2011.08.01

**Published:** 2011-08-20

**Authors:** 帆 杨, 克终 陈, 冠潮 姜, 剑锋 李, 俊 王

**Affiliations:** 100044 北京，北京大学人民医院胸外科 Department of Thoracic Surgery, People's Hospital, Peking University, Beijing 100044, China

**Keywords:** 肺肿瘤, 表皮生长因子受体, 突变, Lung neoplasms, Epidermal growth factor receptor, Mutation

## Abstract

**背景与目的:**

表皮生长因子受体(epidermal growth factor receptor, EGFR)基因突变是肺癌靶向药物疗效的可靠预测指标，因此基因突变的检测具有非常重要的临床意义。本研究建立使用常规实验仪器、高灵敏度、简便的检测表皮生长因子受体突变的方法，以利于临床中快速的检测*EGFR*基因突变。

**方法:**

采用改良的内切酶法富集法检测251例肺腺癌DNA标本中*EGFR*基因外显子19缺失突变和21(L858R)点突变，并与直接测序进行比较。利用混合突变/野生型*EGFR*基因的细胞系测定改良方法的灵敏度。

**结果:**

在251例腺癌标本DNA中使用测序法检测出*EGFR*外显子19突变46例、外显子21突变26例。采用改良的突变体富集法检另外测出外显子19突变78例、外显子21突变57例，总突变率53.8%。灵敏度检测显示对于外显子19和21，新方法的检测灵敏度达0.5%。

**结论:**

本方法具有简便、经济、灵敏度高等特点，便于临床快速筛查非小细胞肺癌病理组织中的*EGFR*基因突变。

## Abstract (en)

**Background and objective:**

Epidermal growth factor receptor (EGFR) gene mutation in lung cancer is a reliable predictor of the efficacy of tyrosine kinase inhibitors, so detection of gene mutation has very important clinical significance. The aim of this study is to optimize conditions for restriction enzyme-based mutant enriched detection of EGFR mutation in lung cancer specimens.

**Methods:**

EGFR somatic mutations identified in DNA specimens from 251 lung adenocarcinomas with modified mutant enriched method and direct sequencing were compared. The sensitivity of the modified method was determined by serial dilution of mutant-carrying lung cancer cells in wild-type cells.

**Results:**

46 deletion mutations in exon 19 and 26 point mutations in exon 21 were found by sequencing, while modified method identified another 78 deletions in exon 19 and 57 substitutions in exon 21 with a mutation rate of 53.8%. Sensitivity of this modified method was 0.5% (mutant/wild type) in serial diluted cells.

**Conclusion:**

The modified restriction enzyme-based method is convenient for clinical *EGFR* mutation screening in non-small cell lung cancer for its simpleness, cost-saving and high sensitivity.

表皮生长因子受体(epidermal growth factor receptor, EGFR)酪氨酸激酶抑制剂(tyrosine kinase inhibitor, TKI)的出现是非小细胞肺癌(non-small cell lung cancer, NSCLC)治疗领域的重大突破。现已确认，肿瘤携带特定的*EGFR*基因突变是药物疗效的可靠预测指标。因此基因突变检测具有非常重要的临床意义。

检测*EGFR*基因突变的方法很多，但绝大多数都需要使用诸如测序仪、实时定量聚合酶链式反应仪、高效液相层析等昂贵的设备，或十分复杂的方法(如单链构象多态性分析，核酸肽锁核酸法等)，难以在医院的普通实验条件下开展。本研究旨在建立使用常规实验仪器、高灵敏度、简便的检测*EGFR*突变方法，以利于国内推广*EGFR*基因突变检测。

## 1 材料与方法

### 1.1 肺癌标本

选取北京大学人民医院保存、经病理证实的肺腺癌手术标本251例，其中231例为新鲜冻存标本，另20例为福尔马林固定的石蜡包埋标本。其中男性135例，女性116例。平均年龄62.1岁(33岁-82岁)，吸烟者103例，不吸烟者148例。Ⅰ期52例，Ⅱ期45例，Ⅲ期98例，Ⅳ期56例。

### 1.2 标本DNA提取

冻存标本取100 mg，机械研磨。石蜡包埋标本取10 μm切片5张，刮入EP管中，以二甲苯脱蜡2次后梯度乙醇水化。DNA提取试剂盒采用天根生化(科技)公司的TIANamp Micro DNA Kit试剂盒，依据厂家提供的说明进行。

### 1.3 EGFR突变检测

方法改良自Asano等^[1]^的方法。原理是标本DNA首先经过特异性消化野生型*EGFR*基因的限制性内切酶切割，再进行聚合酶链式反应(polymerase chain reaction, PCR)扩增。因绝大部分野生型基因已断裂无法作为PCR反应模板，使得扩增产物中突变型基因的比例大大增加。19号外显子缺失突变的鉴定通过PCR产物电泳图中存在较野生型更短的片段(117 bp-108 bp)完成；21号突变鉴定采用特异性切割L858R突变的内切酶二次消化，以存在可被二次消化的PCR产物判定突变(图 1)。具体步骤如下：

**1:**
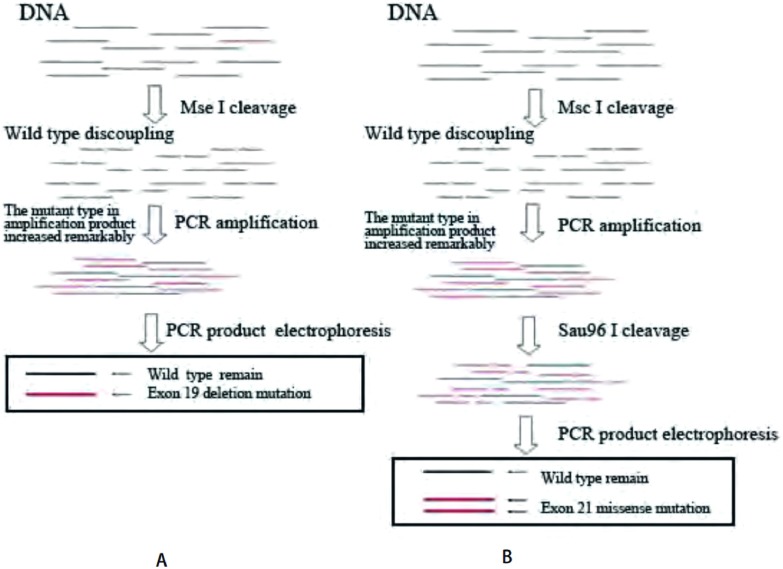
内切酶突变富集法原理图(黑色横线代表野生型*EGFR*基因片段，红色代表突变型片段)。A：外显子19突变检测；B：外显子21突变检测。 Enzyme-based enriched method (Black line represent wild *EGFR* gene segment, red line represent mutant *EGFR* gene segment). A: exon 19; B: exon 21.

#### 1.3.1 EGFR外显子19突变检测方法

标本DNA中加入Mse Ⅰ内切酶2 IU(Fermentas公司)，反应体系20 μL，37 ℃酶切4 h，65 ℃灭活。PCR反应在同管内进行。再加入上下游引物各2 μL、Taq酶(Invitrogen公司)、dNTP、不含镁离子缓冲液及双蒸水至50 uL。上游引物序列5’-AGG GAC TCT GGA TCC CAG AAG GTG-3’，下游引物5’-CCC ACA CAG CAA AGC AGA AAC TCAC-3’。变性94 ℃、退火60 ℃、延长72 ℃，35个循环。PCR产物鉴定采用12%聚丙烯酰胺凝胶恒压电泳，溴化乙锭染色，紫外灯下照相。酶切及PCR反应均在PCR仪上完成。

#### 1.3.2 EGFR外显子21突变检测方法

限制性内切酶消化野生型*EGFR*基因、PCR扩增条件与上一方法相同，内切酶使用Msc Ⅰ(Fermentas公司)。PCR上游引物序列5’-CAG CCA GGA ACG TAC TGG TGA-3’，下游引物5’-TCC CTG GTG TCA GGA AAA TGC T-3’。反应条件与上一方法相同。PCR结束后仍在原管中进行第二轮酶切：加入内切酶Sau96 I 10IU(Fermentas公司)，加缓冲液及双蒸水至体系60 μL。37 ℃酶切4 h，PCR产物鉴定采用聚丙烯酰胺凝胶电泳与上一方法相同。

### 1.4 检测灵敏度(阈值)鉴定

将野生型*EGFR*基因的A549细胞(本实验室保存)与携带外显子19缺失突变HCC827人肺癌细胞(购自美国模式培养物集存库)，或21号外显子L858R突变H1975细胞(购自中国医学科学院细胞库)，根据报道^[2, 3]^的*EGFR*基因拷贝数3.4、36.0和3进行系列稀释后，提取基因组DNA，使得其中突变型与野生型*EGFR*基因的比例分别为：1:1、1:10、1:20、1:50、1:100、1:200、1:400和1:1, 000。分别按上述检测方法进行基因突变的检测。

### 1.5 测序

所有采用内切酶法检测的标本DNA采用相同引物进行35个循环PCR扩增，50 μL体系，反应条件同前。扩增产物送华大基因公司进行上、下游引物双向测序。序列分析采用Mutation Surveyor软件。

## 2 结果

### 2.1 采用优化的内切酶富集法检测251例标本与直接测序法检测结果相比较

相同的251例标本DNA，采用直接测序，检测到外显子19和21突变72例，突变率28.7%，采用本研究的内切酶法检测到135例，突变率53.8%，所有测序法发现的突变均检测到之外，又检测到突变63例(表 1)。

**1:** 标本*EGFR*基因突变检测测序法与内切酶法结果比较 Comparison of modified restriction enzyme-based detection and direct sequencing for *EGFR* mutation

	Enzyme-based enriched method	Direct sequencing method	Enriched(+) and Sequencing(-)	Enriched(-) and Sequencing(+)
Exon 19	78 (31.1%)	46 (18.3%)	32	0
Exon 21	57 (22.7%)	26 (10.4%)	31	0
Total	135 (53.8%)	72 (28.7%)	63	0

### 2.2 灵敏度测试

当突变型基因与野生型*EGFR*基因比例大于1：200时(0.5%)时，即可见到突变的特异性条带，表明检测阈值为0.5%(突变型/野生型基因)。PCR产物电泳图见图 2。

**2:**
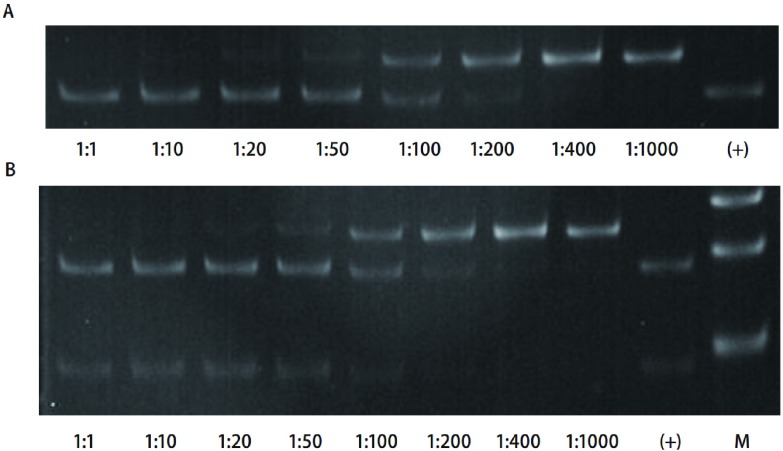
内切酶突变富集法灵敏度测试。A：外显子19；B：外显子21。 Sensativity measurement of modified restriction enzyme-based enriched method. A: Exon 19; B: Exon 21.

## 3 讨论

EGFR TKI的出现为晚期NSCLC提供了全新的治疗途径。研究^[4]^发现，EGFR TKI的疗效与肿瘤细胞中携带着特定*EGFR*基因突变显著相关，特别是占全部突变约90%的外显子19缺失突变和外显子21的L858R点突变，有效率达70%以上，反之则极少有效。因此，*EGFR*突变的检测成为了临床用药的重要参考指标。随着吉非替尼和紫杉醇/卡铂一线治疗对比的IPASS研究^[5]^和西班牙厄洛替尼一、二线治疗研究^[6]^的发表，EGFR TKI甚至成为了携带EGFR突变的晚期患者的一线治疗，彻底打乱了原有的传统化疗流程，同时也把*EGFR*突变检测的重要性提升到了前所未有的高度。

最初采用的EGFR突变检测方法是PCR扩增后的直接测序，因为能够直接鉴定突变的类型，测序法也成为了金标准。但是，这种方法的灵敏度不高，要求肿瘤细胞在标本中的比例要大于25%^[7]^，但临床标本取材也往往混杂大量的正常细胞背景。为保证测试的准确性，美国EGFR检测协作组建议标本必须在病理科医师的协助下选取石蜡切片中肿瘤细胞50%-70%区域进行细胞富集^[8]^。

除测序法外，将近十余种的检测方法被设计出来，目的是灵敏、准确的检测到突变^[9]^。包括实时定量聚合酶链式反应(TaqMan)法^[10]^、变性高效液相层析(DHPLC)^[11]^、高分辨溶解曲线分析^[12]^、单链构象多态性分析(SSCP)^[13]^、核酸肽锁核酸法^[14]^、杂交环电泳率分析法^[15]^、扩增受阻突变体系(ARMS)^[16]^等。这些方法或需要昂贵的设备，或采用步骤繁琐对实验条件要求很高，难以在常规实验条件下开展。相比之下本研究的方法简单易行，除普通PCR仪和凝胶电泳设备外无需其它昂贵设备，而且检测阈值达到0.5%(表 2)。

**2:** 各种*EGFR*基因突变检测方法的比较 Comparison of the detection methods of *EGFR* gene mutations

Method	Sensibility	Special instrument	Price	Shortage
Direct sequencing	25%	Sequenator	High	Low sensibility, long detection cycle
TaqMan PCR	10%	Real-time PCR	High	Strict experimental conditions, cost expensive
DHPLC	1%	High-performance liquid chromatograph	High	Special instrument
SSCP	10%	None	Low	Low sensibility, difficult temperature control
PNA-LNA	1%	Real-time PCR	Higher	Speicial probe, cost expensive
Loop-hybrid	7.5%	None	Higher	Probe hybridation, complicate
ARMS	1%	Real-time PCR	Highest	Cost expensive, strict experiment condition
Enzyme-based enriched method	0.5%	None	Low	

对于所有建立在PCR反应上的检测方法，尽可能避免污染是关键。除严格设定阴性、阳性对照，分区进行PCR反应和电泳外，本研究改良的主要工作就是简化步骤、减少污染可能。首先，原方法采用了二轮PCR扩增的方法，虽然灵敏度极高，可检测出0.5‰(1:2, 000)的携带突变的细胞^[1]^，但步骤繁琐，需要对PCR产物进行纯化、酶切，大大增加了污染的机会。本研究把PCR扩增减少到一轮。测试表明，改良的方法仍可以保持0.5%的检测阈值，满足检测的要求。其次针对PCR容易产生污染的问题，通过优化反应条件，主要是匹配各步骤的离子强度，将所有反应尽可能在同一个PCR管中完成，不断加入试剂。仅在进行电泳时涉及移出PCR产物，对于外显子21的点突变检测，由于只有L858R突变体已被酶切，其对后续标本的污染可能性非常小。

我们在256例腺癌中共检测出EGFR外显子19突变78例，外显子21突变57例，突变率53.8%，与IPASS研究中采用ARMS方法检测的外显子19和21的突变率57.4%类似^[6]^。相同的DNA标本采用测序法和新方法检测对比，本研究优化的内切酶富集法灵敏，没有漏检，却相比直接测序多检测到将更多的突变(53.8% *vs* 28.7%)。由于本实验的方法较测序法更加灵敏，因此新方法的特异性和准确性无法得到验证，仅能通过与IPASS研究中检测结果对比间接表明结果可信，这是本研究的不足之处。此外，本方法无法检测18、20外显子等其它罕见突变，也是其局限性所在。另外需要指出的是本研究使用的标本没有经过病理科医师的选择，肿瘤细胞比例很可能达不到美国EGFR检测协作组建议的标准。采用这种“不达标”标本的目的是比较测序法和改良突变富集法的灵敏度，测序法出现假阴性不但不能否定测序，反而更强调了美国EGFR检测协作组建议的重要性。

综上，本研究优化了新的肺癌标本中*EGFR*基因突变检测方法，其最大优势在于不需要特殊的昂贵仪器，且具体的实验技术较简单，耗时短，试剂简单费用低。这对于国内大多数医院而言，都具有可操作性，有推广的价值。
